# The efficiency and safety of fascia iliaca block for pain control after total joint arthroplasty: A meta-analysis

**DOI:** 10.1097/MD.0000000000006592

**Published:** 2017-04-14

**Authors:** Peng Zhang, Jifeng Li, Yuze Song, Xiao Wang

**Affiliations:** Department of Orthopedics, Huaihe Hospital, Henan University, Henan, China.

**Keywords:** fascia iliaca block, meta-analysis, pain control, total hip arthroplasty, total knee arthroplasty

## Abstract

**Background:**

This meta-analysis aimed to perform a meta-analysis including randomized controlled trials (RCTs) to assess the efficiency and safety of fascia iliaca block (FIB) for pain control in patients undergoing total joint arthroplasty (TJA).

**Methods:**

A systematic search was performed in Medline (1966–2017.03), PubMed (1966–2017.03), Embase (1980–2017.03), ScienceDirect (1985–2017.03) and the Cochrane Library. Study evaluated the efficiency and safety of FIB in TJA was selected. Meta-analysis was performed using Stata 11.0 software.

**Results:**

Five randomized controlled trials (RCTs) including 270 patients met the inclusion criteria. The present meta-analysis indicated that there were significant differences between groups in terms of visual analog scale (VAS) score at 12 hours (SMD = −0.544, 95% CI: −0.806 to −0.281, *P* = .000) and 24 hours (SMD = −0.519, 95% CI: −0.764 to −0.273, *P* = .000), morphine equivalent consumption at 12 hours (SMD = −0.895, 95% CI: −1.164 to −0.626, *P* = .000) and 24 hours (SMD = −0.548, 95% CI:−0.793 to −0.303, *P* = .000). In addition, fewer adverse side effect was identified in FIB groups (RD = −0.139, 95% CI: −0.243 to −0.034, *P* = .009).

**Conclusion:**

The application of fascia iliaca block could significantly reduce VAS scores and morphine consumption at 12 and 24 hours following total knee and hip arthroplasty. In addition, there were fewer adverse effects in FIB groups. Due to the limited quality of the evidence currently available, higher quality RCTs are required.

## 1 Introduction

Total knee arthroplasty (TKA) and total hip arthroplasty (THA) are well-known popular surgical procedures for treatment of the degenerative disorders and traumatic diseases. However, patients often suffer moderate to severe postoperative pain.^[1–3]^ Adequate postoperative pain control is crucial for early ambulation and satisfied functional outcomes were achieved following early postoperative rehabilitation. Moreover, optimal pain management can reduce duration of hospitalization and the risk of adverse events, such as deep vein thrombus (DVT) and pulmonary embolism (PE).

Postoperative pain control is still a subject for a few decades and remains controversial. Numerous strategies have been implemented to including local anesthetic infiltration, systemic opioids, femoral nerve block, and spinal analgesia.^[4–7]^ Although these methods have been shown to relive pain in previous studies, substantial opioids consumption usually happens which may lead to adverse side effects such as nausea, vomiting, respiratory depression, hypotension, and other systemic reaction, which causes a delayed recovery and poor quality of life for patients undergoing total joint arthroplasty (TJA).^[6,8]^

Peripheral nerve block is commonly used for postoperative analgesia following total knee and hip arthroplasty. Femoral nerve block is considered an effective method; however, it was criticized for the risk of nerve injury and weakness in quadriceps muscle strength. Paul et al^[9]^ reported that there is an increased risk of falls for patients who received femoral nerve block. Recently, fascia iliaca block (FIB) was proposed as a popular analgesic technique which involves local infiltration anesthesia under the fascia of the iliacus muscle. The method depends on the local anesthetics spread beneath the fascia to block the peripheral nerve.

Currently, the application of fascia iliaca block in total knee and hip arthroplasty was seldom reported. Thus, there was no reliable evidence regarding the analgesic and morphine-sparing effect for fascia iliaca block. Therefore, we perform a meta-analysis including published clinical research to assess the efficiency and safety of FIB for pain control in patients undergoing total knee and hip arthroplasty.

## 2 Methods

### 2.1 Search strategy

**Figure 1:**
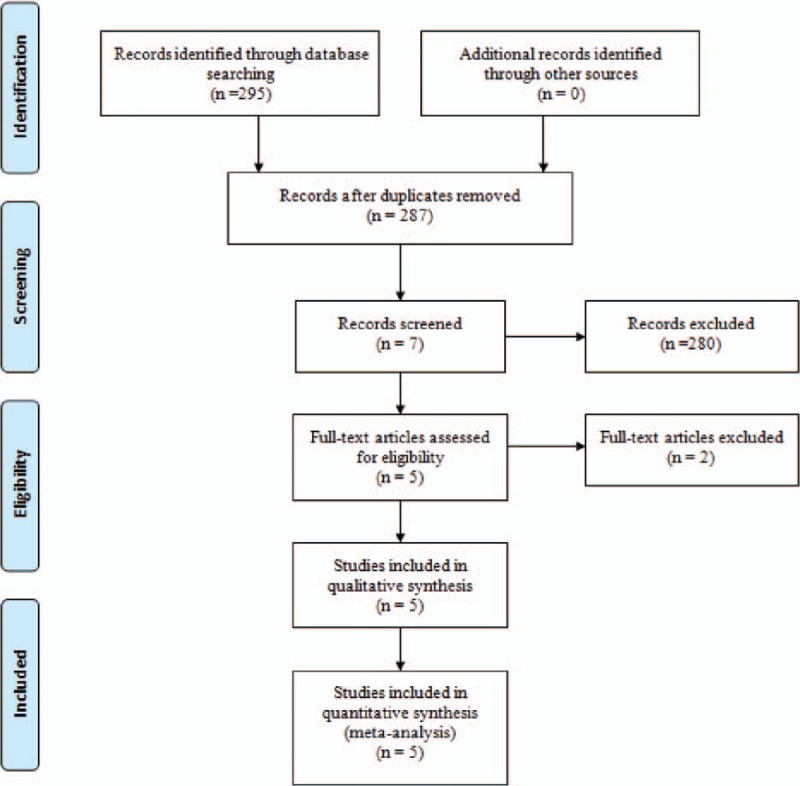
Search results and the selection procedure.

Electronic databases were systemically searched including Embase (1980–2017.03), Medline (1966–2017.03), PubMed (1966–2017.03), ScienceDirect (1985–2017.03), web of science (1950–2017.03), and Cochrane Library for potential relevant studies. Reference lists of all the potential included studies and relevant reviews were hand-searched for any additional trials. No restrictions were imposed on language. The search terms were as follows: “total knee replacement OR arthroplasty,” “total hip replacement OR arthroplasty,” “fascia iliaca block,” and “pain control” were used in combination with Boolean operators AND or OR. The retrieval process is presented in Fig. 1.

### 2.2 Inclusion and exclusion criteria

Studies were considered eligible if they met the following criteria: (1) published clinical randomized control trails (RCTs) and non-RCTs; (2) patients undergoing TKA or THA, experiment group received FIB for pain control and control group received no block; (3) the primary outcomes, including visual analogue scale (VAS) scores and morphine consumption at different times. Secondary outcomes included length of hospital stay and postoperative adverse effects such as the incidence of nausea, vomiting, and falls. Studies would be excluded from current meta-analysis for incomplete data, case reports, conference abstract, or review articles. All analyses were based on previous published studies; thus, no ethical approval and patient consent are required.

### 2.3 Selection criteria

Two authors independently reviewed all the abstracts of the potential studies identified by the above searches. After an initial decision, the full text of the studies that potentially met the inclusion criteria were reviewed and the final decision was made. A senior reviewer is consult in the case of disagreement regarding which studies to include.

### 2.4 Date extraction

A standard form for date extraction is printed for date extraction. Two authors independently extracted the relevant data from the included articles. Details of incomplete data of included studies are obtained by consulting the corresponding author. Following data was extracted: First author names, published year, study design, comparable baseline, anesthesia methods, and dosage and type of anesthetic drug for FIB. Outcome parameters included VAS scores at different periods, the cumulative morphine consumption, length of hospital stay, and morphine-related adverse effects. Other relevant data was also extracted from individual studies.

### 2.5 Quality assessment

Quality assessment of the included studies was assessed by 2 authors independently. Modified Jadad score (7-points scale) which was based on Cochrane Handbook for Systematic Reviews of Interventions is used for assessment of RCTs. Studies which scores greater than 4 points was considered high quality. We conducted “risk of bias” table including the following key points: random sequence generation, allocation concealment, blinding, incomplete outcome data, free of selective reporting and other bias, each item was recorded by “Yes,” “No,” or “Unclear.” The Methodological Index for Non-Randomized Studies (MINORS) scale was used to evaluate non-RCTs with scores ranging 0 to 24. A consensus is reached through a discussion.

The qualities of evidence of main outcomes in present meta-analysis were evaluated using the Recommendations Assessment, Development and Evaluation (GRADE) system including the following items: risk of bias, inconsistency, indirectness, imprecision, and publication bias. The recommendation level of evidence is classified into the following categories: (1) high, which means that further research is unlikely to change confidence in the effect estimate; (2) moderate, which means that further research is likely to significantly change confidence in the effect estimate and may change the estimate; (3) low, which means that further research is likely to significantly change confidence in the effect estimate and to change the estimate; and (4) very low, which means that any effect estimate is uncertain.

### 2.6 Data analysis and statistical methods

All calculations were performed using Stata 11.0 software (The Cochrane Collaboration, Oxford, United Kingdom). Statistical heterogeneity was assessed based on the value of *P* and *I*^2^ using the standard chi-square test. When *I*^2^>50%, *P* < 0.1 was considered to be significant heterogeneity, the random-effect model was performed for meta-analysis. Otherwise, the fixed-effect model was used. If possible, sensibility analysis is conducted to explore the origins of heterogeneity. The results of dichotomous outcomes were expressed as risk difference (RD) with 95% confidence intervals (CIs). For continuous various outcomes, mean difference (MD) and standard mean difference (SMD) with a 95% confidence intervals (CIs) was applied for assessment.

## 3 Results

### 3.1 Search result

A total of 295 studies were preliminarily reviewed. By screening the titles and reading the abstracts and entire contents, 290 reports were excluded from present meta-analysis following inclusion criteria. No gray reference was included. Finally, 5 randomized controlled trials (RCT)^[10–14]^ which had been published between 2007 and 2016 were enrolled in present meta-analysis and includes 137 participates in the FIB groups and 133 patients in the control groups.

### 3.2 Study characteristics

The sample size of the included studies ranged from 32 to 85. All of them evaluated the efficiency and safety of FIB for pain control in TJA. Experimental groups received FIB, whereas control groups no block. There is a variation in dosage and type of the anesthetic drugs in FIB. Four studies^[11–14]^ performed general anesthesia and 1^[10]^ applied lumbar subarachnoid block. Three^[10–12]^ studies reported that total joint arthroplasties was performed by same surgeons. All articles reported that patient-controlled analgesia was used for concomitant pain management. Only Desmet et al^[12]^ performed a sample size calculation. All of them suggest the outcomes for at least 95% of the patients. The follow-up period ranged from 1 to 6 months.

### 3.3 Risk of bias assessment

Demographic characteristics, the details about the included studies are summarized in Table 1. Modified Jadad score which was based on Cochrane Handbook for Systematic Reviews of Interventions is used for assessment of RCTs (Table 2). All of the RCTs^[10–14]^ reported a clear inclusion and exclusion criteria and suggest a methodology of randomization, all of them demonstrated that randomization sequence was generated by computer. Three of them^[10,12,13]^ reported allocate concealment was achieved by sealed envelopes. Double blinding was provided in 2 RCTs^[10,14]^. One^[14]^ of them had attempted to blind assessors. Each risk of bias item is presented as the percentage across all included studies, which indicates the proportion of different levels of risk of bias for each item (Table 3). All RCTs provided complete outcome data. Only 1^[12]^ of them performed intent–to-treatment analysis thus a potential risk for type II statistical error would exist.

**Table 1:**
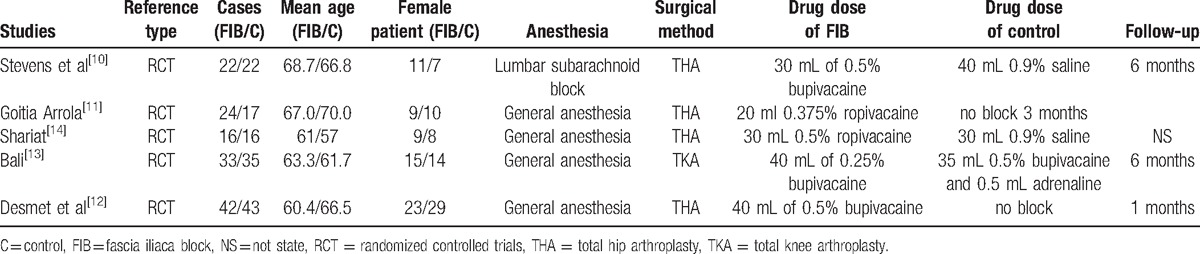
Trials characteristics.

**Table 2:**
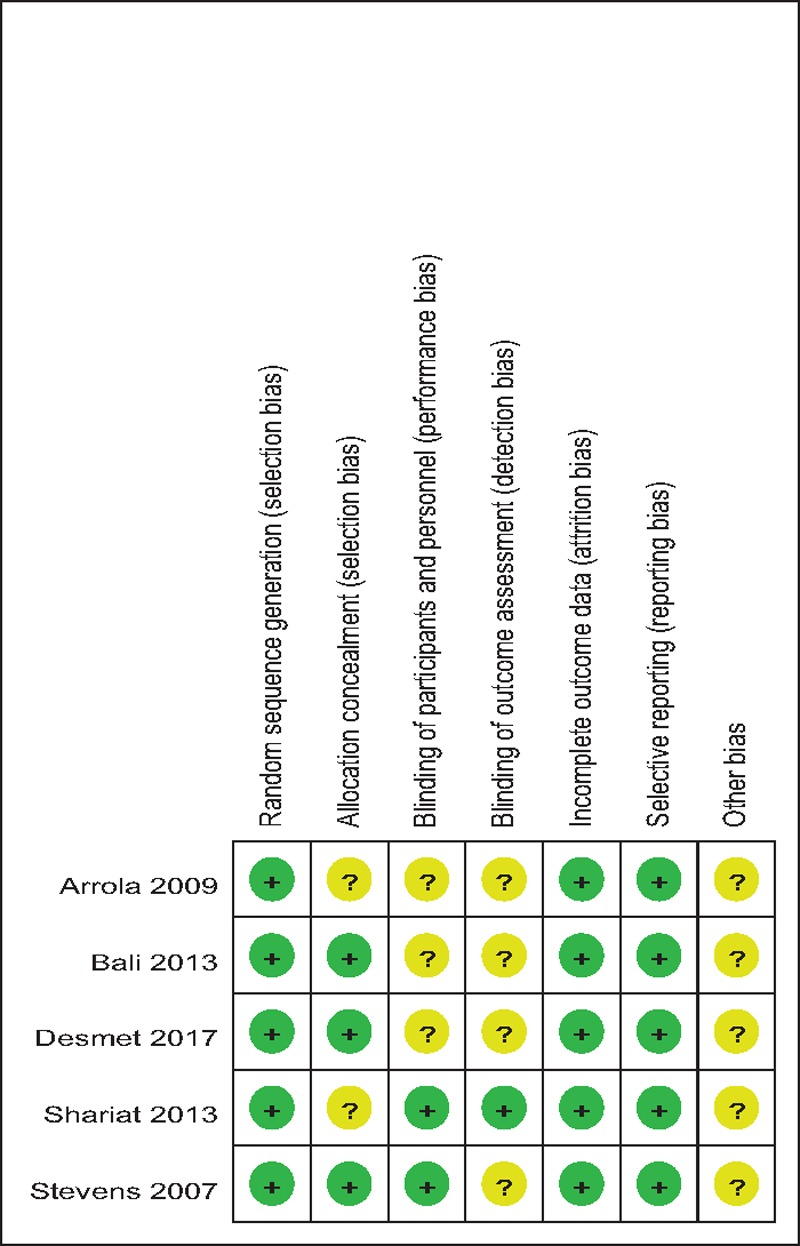
Methodological quality of the randomized controlled trials.

**Table 3:**
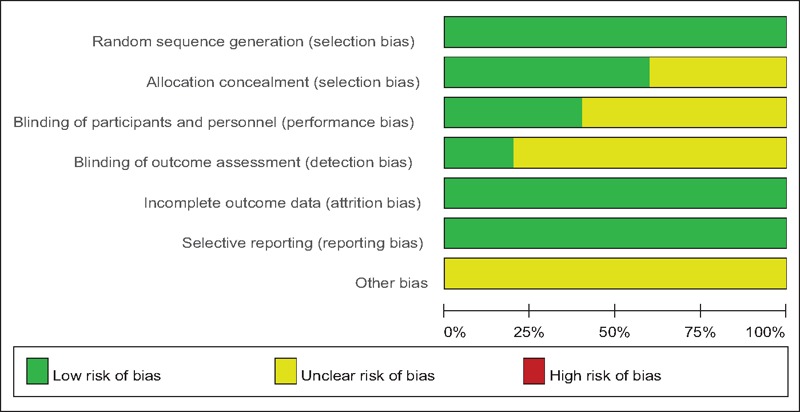
Risk of bias of included RCTs. RCT = randomized controlled trials.

### 3.4 Outcomes for meta-analysis

#### 3.4.1 VAS scores at 12 hours

**Figure 2:**
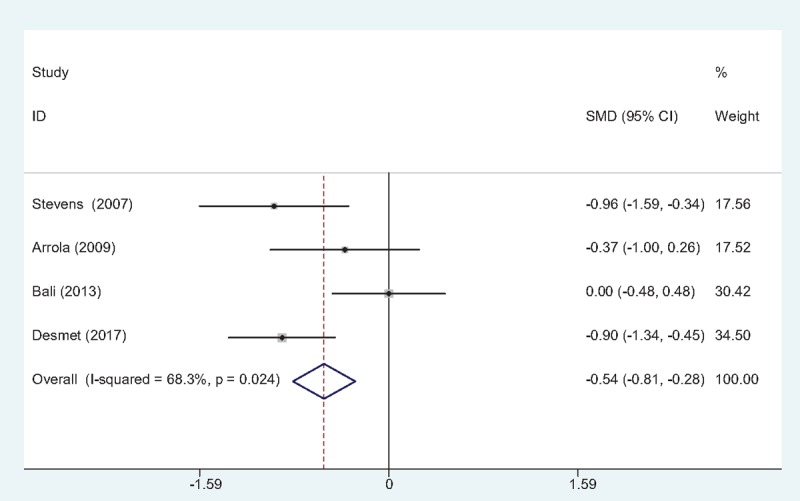
Forest plot diagram showing VAS scores at 12 h following TJA.TJA = total joint arthroplasty, VAS = visual analog scale.

Four studies^[10–13]^ reported VAS scores at 12 hours following TJA. Statistical heterogeneity was observed in present meta-analysis (χ2 = 9.46, df = 3, *I*^2^ = 68.3%, *P* = .024); therefore, a random-effects model was applied. We found that there was significant difference between the FIB and control groups regarding the VAS scores at 12 hours (SMD = –0.544, 95% CI: –0.806 to –0.281, *P* = .000; Fig. 2).

#### 3.4.2 VAS scores at 24 hours

**Figure 3:**
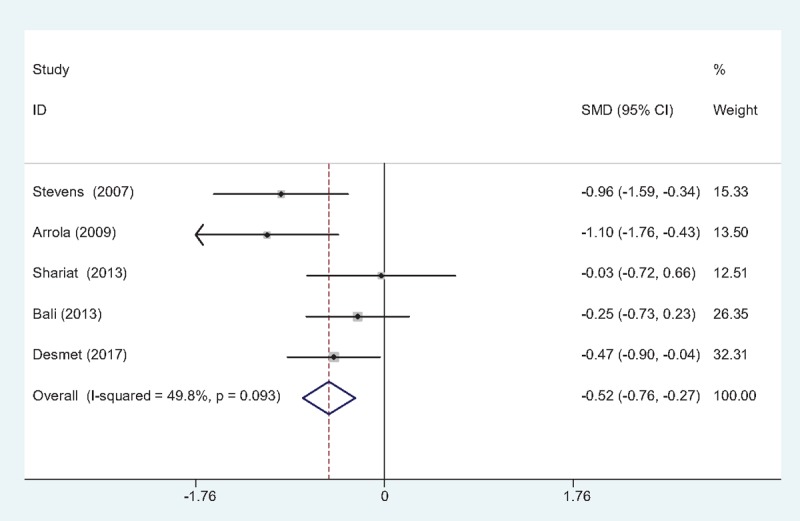
Forest plot diagram showing VAS scores at 24 h following TJA. TJA = total joint arthroplasty, VAS = visual analog scale.

Five studies^[10–14]^ reported VAS scores at 24 hours following TJA. There was no significant heterogeneity (χ2 = 7.97, df = 4, *I*^2^ = 49.8%, *P* = .093); therefore, a fixed-effects model was used. The result of meta-analysis showed that there was significant difference between the FIB groups and control groups regarding the VAS scores at 24 hours (SMD = –0.519, 95% CI: –0.764 to –0.273, *P* = .000; Fig. 3).

#### 3.4.3 Morphine consumption at 12 hours

**Figure 4:**
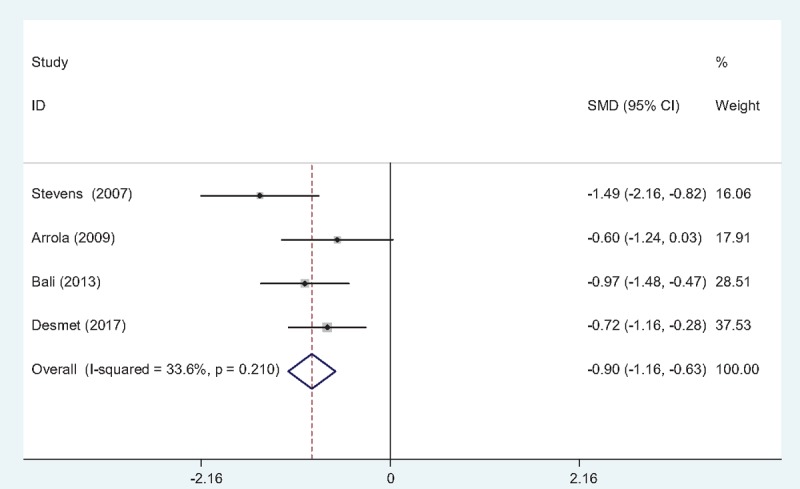
Forest plot diagram showing morphine consumption at 12 h following TJA. TJA = total joint arthroplasty.

Morphine consumption at postoperative 12 hours was presented in 4 studies^[10–13]^ following TJA. There was no significant heterogeneity (χ2 = 4.52, df = 3, *I*^2^ = 33.6%, *P* = .210) and a fixed-effects model was used. The present meta-analysis showed that there was significant difference between the FIB and control groups in terms of morphine consumption at postoperative 12 hours (SMD = –0.895, 95% CI: –1.164 to –0.626, *P* = .000; Fig. 4).

#### 3.4.4 Morphine consumption at 24 hours

**Figure 5:**
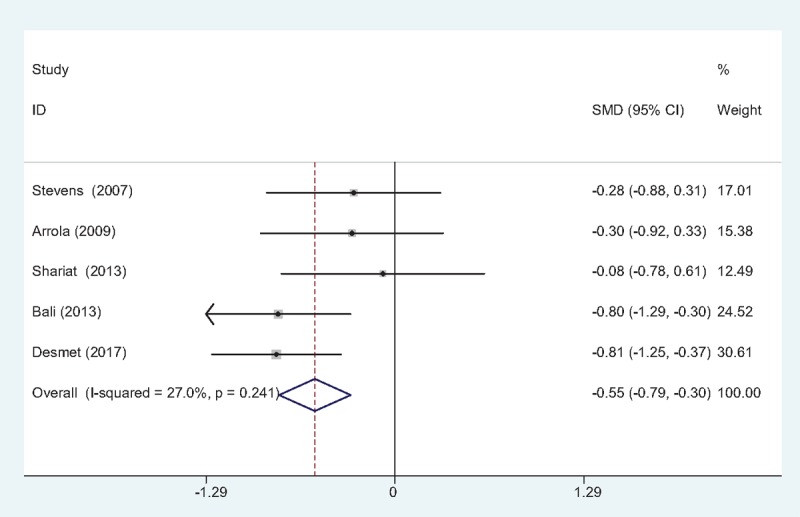
Forest plot diagram showing morphine consumption at 24 h following TJA. TJA = total joint arthroplasty.

Five studies^[10–14]^ provided morphine consumption at postoperative 24 hours following TJA. No significant heterogeneity was found (χ2 = 5.48, df = 4, *I*^2^ = 27.0%, *P* = .241); therefore, a fixed-effects model was used. Meta-analysis revealed that there was significant difference between the FIB and control groups in terms of morphine consumption at postoperative 24 hours (SMD = –0.548, 95% CI: –0.793 to –0.303, *P* = .000; Fig. 5).

#### 3.4.5 Length of hospital stays (LOS)

**Figure 6:**
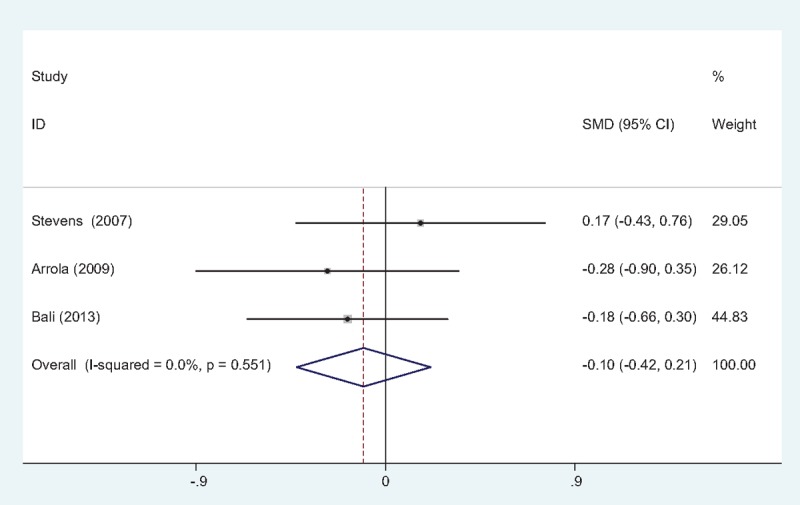
Forest plot diagram showing length of hospital stay following TJA. TJA = total joint arthroplasty.

Three studies^[10,11,13]^ reported the length of hospital stays between groups. No significant heterogeneity was identified in the pooled results; therefore, a fixed-effects model was used (χ2 = 1.19, df = 2, *I*^2^ = 0%, *P* = .551). There was no significant difference between the 2 groups in LOS (SMD = –0.105, 95% CI: –0.424 to 0.214, *P* = .520; Fig. 6).

#### 3.4.6 The occurrence of nausea and vomiting

**Figure 7:**
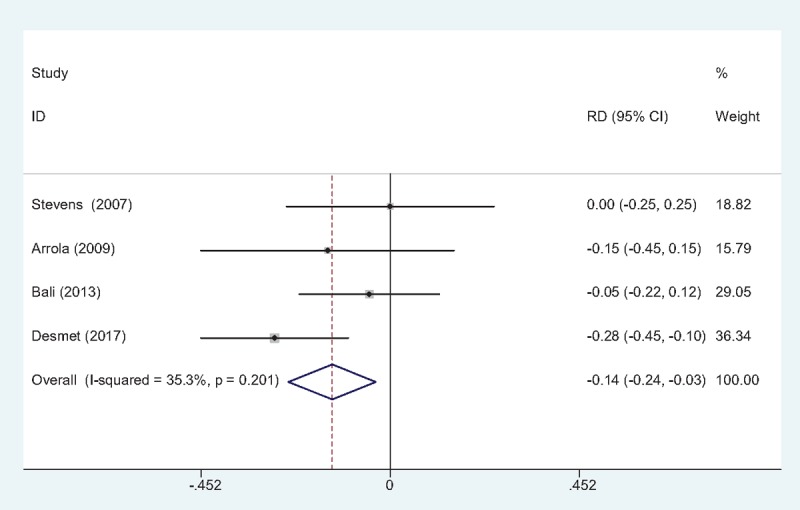
Forest plot diagram showing incidence of nausea and vomiting following TJA. TJA = total joint arthroplasty.

The occurrence of nausea and vomiting was showed in 4 studies.^[10–13]^ No significant heterogeneity among these studies was found; therefore, a fixed-effects model was used (χ2 = 4.64, df = 3, *I*^2^ = 35.3%, *P* = .201). There was significant difference between the 2 groups in the incidence of nausea and vomiting (RD = –0.139, 95% CI: –0.243 to –0.034, *P* = .009; Fig. 7).

#### 3.4.7 The occurrence of falls

**Figure 8:**
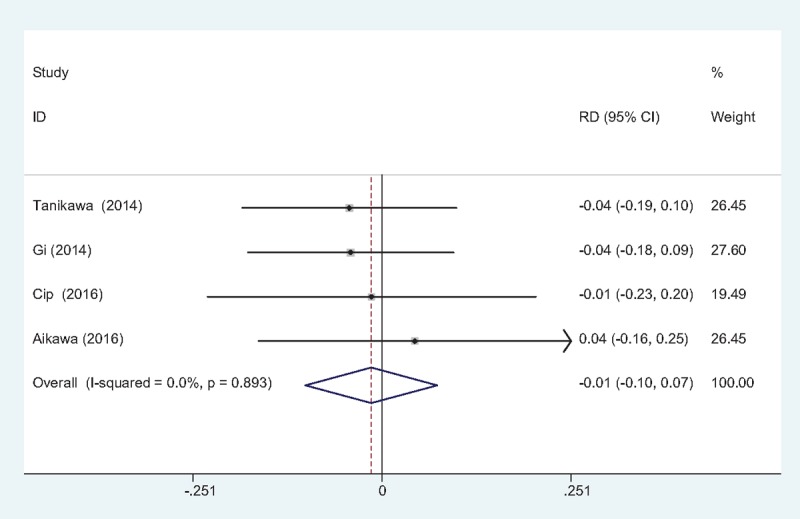
Forest plot diagram showing incidence of falls following TJA. TJA = total joint arthroplasty.

Three studies^[10–13]^ reported the incidence of falls. No statistical heterogeneity was observed and a fixed-effects model was applied (χ2 = 0.86, df = 2, *I*^2^ = 0%, *P* = .649). The present meta-analysis indicated that there is no significant difference regarding the frequency of falls between groups (RD = 0.000, 95% CI: –0.050 to 0.051, *P* = .989; Fig. 8).

#### 3.4.8 Publication bias

**Figure 9:**
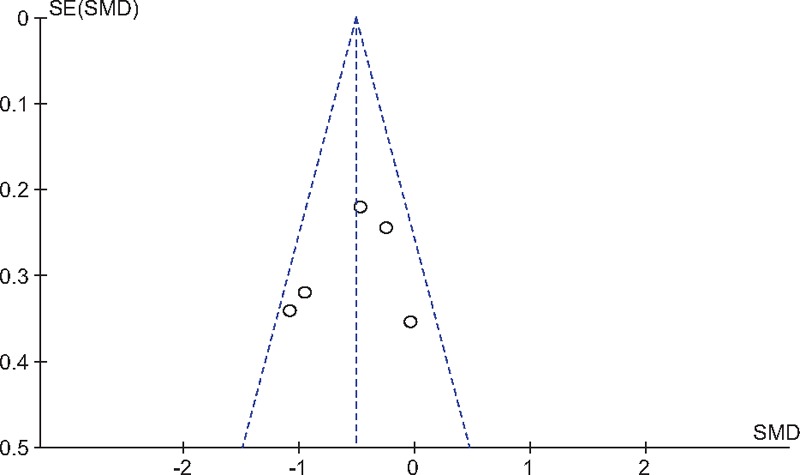
Funnel plot of VAS scores at 24 h following TJA. TJA = total joint arthroplasty, VAS = visual analog scale.

**Figure 10:**
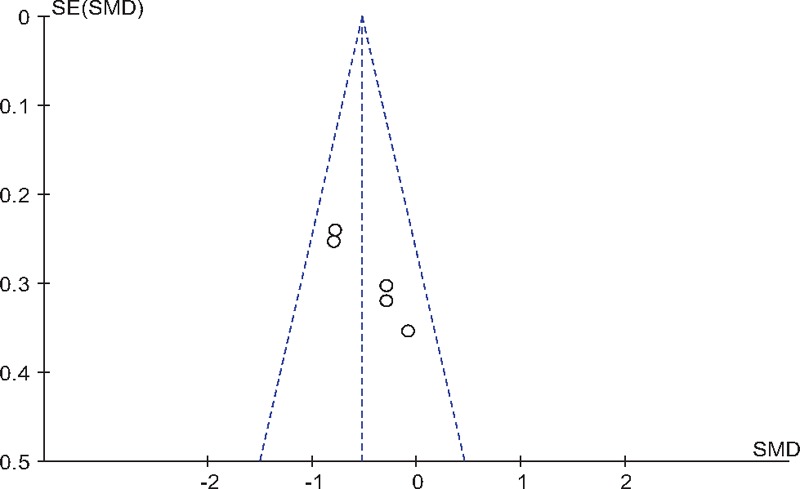
Funnel plot of morphine consumption at 24 h following TJA. TJA = total joint arthroplasty.

As all studies VAS scores at 24 hours after TJA, publication bias was assessed and presented in Fig. 9. Funnel plots were symmetrical and low risk of publication bias was showed. Figure 10 assessed the publication bias of morphine consumption 24 hours following TJA, and showed low risk either. However, publication bias could not be excluded as the reliability of this kind of assessment was weak especially when a low number of studies were included.

## 4 Discussion

To the best of our knowledge, this study is the first meta-analysis to assess the efficiency and safety of FIB for pain control in patients undergoing total knee and hip arthroplasty from randomized controlled trials. The most important finding of the meta-analysis was that the application of fascia iliaca block could significantly reduce the VAS scores and morphine consumptions at 12 and 24 hours after TJA. Moreover, there is a decreased risk of nausea and vomiting in FIB groups compared controls. All outcomes in this meta-analysis were evaluated using the GRADE system. The evidence quality for each outcome was high to moderate (Table 4) which means that further research is likely to significantly change confidence in the effect estimate and may change the estimate.

**Table 4:**
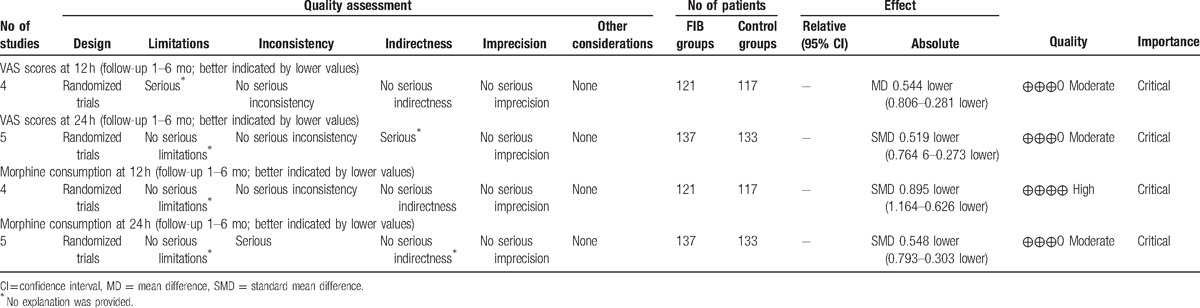
The GRADE evidence quality for main outcome.

With the ageing population, the occurrence of osteoarthritis is increasing, and TJA is a popular treatment. Pain control following TKA can be very challenging. Optimal analgesia may shorten hospital stays and result in decreased risks of deep vein thrombosis (DVT) and pulmonary embolism (PE). Furthermore, early rehabilitation exercise contributes to a satisfied sufficient functional recovery. Multimodal techniques featuring peripheral nerve blocks have shown superior efficacy for pain relief in TJA. Femoral nerve block (FNB) has been widely applied in joint arthroplasty surgery and demonstrated outstanding analgesia effect pain and significantly decreased morphine consumption.^[15,16]^ However, FNB was criticized for the risk of femoral nerve injury and a potential for injury to the femoral vessels.^[17]^ It has been reported that the FIB could avoid such complications and provide equivalent analgesia by anesthetizing the femoral nerve remotely from important neurovascular structures. FIB was considered an alternative choice for pain control. Brisbane et al^[18]^ found that there is no significant difference in pain scores and opioids consumption in different periods between FIB and FNB in patients undergoing TKA; thus, FIB is as effective as FNB as part of a multimodal anesthetic regimen for TKA. The present meta-analysis indicated that FIB could significantly reduce VAS scores at 12 and 24 hours following total knee and hip arthroplasty.

Total joint arthroplasty is usually associated with severe pain in 60% and moderate pain in 30% of patients, especially in the first 48 hours and after postoperative mobilization pain remains intense.^[19]^ Additional opioids, including oral and patient-controlled analgesia (PCA) administration, were applied as concomitant pain control. Opioid consumption is considered an objective method to measure pain. Opioids-related adverse effects, such as nausea, vomiting, respiratory depression, and hypotension, were frequently reported in previous studies.^[20,21]^ Besides the side effects described above, drug dependence is also an important issue that should be considered. Minimizing opioid consumption would improve patient satisfaction and expedite mobilization and rehabilitation. The application of a local anesthetic nerve block is recommended by the UK National Institute of Health and Care Excellence as part of an opioid sparing strategy.^[22]^ The FIB has been proposed to block the femoral nerve, the obturator nerve, and the lateral cutaneous nerves.^[23,24]^ There are theoretical advantages in the management of the postoperative pain following TJA. Foss et al^[25]^ showed an opioid sparing effect and superior pain relief in management of hip fracture pain by fascia iliaca block in a randomized, placebo-controlled trial. McMeniman reported that fascia iliaca block is as effective as femoral nerve block as part of a multimodal anesthetic regimen for TKA. The present meta-analysis showed that the application of FIB could significantly reduce morphine consumption at 12 and 24 hours following total knee and hip arthroplasty.

Nausea and vomiting are common side effects that are frequently associated with intravenous or intrathecal morphine. Sufficient anaesthetic techniques can reduce morphine consumption and subsequently decrease the risk of opioids-related complications. The incidence of nausea and vomiting is 20/101 in FIB groups compared 34/81 in controls. The present meta-analysis indicated that there was a decreased risk of nausea and vomiting in FIB groups compared controls. Maybe morphine-sparing effect leads to the results. Considering that only 5 studies were included in our meta-analysis, and thus we did not perform investigation on dose-dependence. Large sample sizes from high-quality RCTs are needed. Motor weakness and risk of falls following peripheral nerve block are potential problems which can delay mobilization after TJA.^[26]^ We found no increased risk of falls in present meta-analysis.

The present meta-analysis exists some limitations that should be noted. (1) Only 5 studied were included in present meta-analysis, although all of them are recently published RCTs, the sample size are relatively small; (2) functional outcome is an important parameter; due to the insufficiency of relevant data, we fail to perform a meta-analysis. (3) Dose of anesthetics are varied and concomitant pain management regime differs from each other, which may influence the results of the meta-analysis; (4) The duration of follow up is relatively short which leads to underestimating complications. (5) Publication bias in present meta-analysis may influence the results.

Despite the limitations above, this is the first meta-analysis from randomized controlled trials to assess the efficiency and safety of FIB for pain control following total knee and hip arthroplasty. Long-term of high-quality RCTs were needed to explore the functional outcome of the knees and other adverse effects.

## 5 Conclusion

The application of fascia iliaca block could significantly reduce VAS scores and morphine consumption at 12 and 24 hours following total knee and hip arthroplasty. In addition, there were fewer adverse effects in FIB groups. Due to the limited quality of the evidence currently available, higher quality RCTs are required.
